# IL-2 and IL-15 augment HBV therapeutic vaccination and PD1 blockade for functional cure in the AAV-HBV mouse model

**DOI:** 10.3389/fimmu.2025.1562107

**Published:** 2025-07-16

**Authors:** Gavin Lewis, Kirsten Malo, Thomas Rowland, Jenaya Hooks, Hao Yuan Liu, Sonam Popli, George Kukolj, Craig S. Pace

**Affiliations:** Infectious Diseases Discovery, Infectious Diseases and Vaccines, Johnson & Johnson Innovative Medicine, Brisbane, CA, United States

**Keywords:** hepatitis B virus, antiviral agents, small interfering RNA, vaccine immunology, immunomodulation, functional cure

## Abstract

**Introduction:**

Prevalence of chronic hepatitis B virus (HBV) infection remains a major global health issue. Research into a cure has focused on finite combinatorial interventions that aim to reduce HBV surface antigen (HBsAg), suppress virus specific immune tolerance, and induce an adaptive response that functionally controls the virus.

**Methods:**

In C57BL/6 mice transduced with adeno-associated virus encoding the HBV genome, which replicate HBV and persistently express HBsAg at 10^4^ IU/mL or higher, a combination of small interfering RNA (siRNA) knockdown of HBsAg expression followed by immunization with a self amplifying RNA therapeutic HBV vaccine failed to establish HBV control. Using this *in vivo* murine model, we screened for immunomodulatory agents added after HBV siRNA knockdown, and in combination with therapeutic vaccination, that may enhance the HBV adaptive immune response to control HBV.

**Results/Discussion:**

In mice with very high levels of HBsAg (10^4^–10^5^ IU/mL), levels that are observed clinically during standard HBV therapy and that were brought low (10^2^ IU/mL) by HBV siRNA pre-treatment prior to therapeutic vaccination, PDL1 blockade in combination with stabilized cytokines IL-2 or IL-15 led to immune control of HBsAg in vaccinated animals.

## Introduction

Chronic hepatitis B virus (HBV) infection (CHB) is a global public health concern with an estimated 250 million people living with the disease worldwide, resulting in premature death from liver cirrhosis or hepatocellular carcinoma in 15%–25% of patients (1, 2). The ultimate therapeutic goal is functional cure (FC), defined as sero-clearance of HBV surface antigen (HBsAg) and undetectable HBV DNA sustained for at least 6 months off treatment. The current standard of care (SOC), nucleos(t)ide analogs (NAs), and/or pegylated interferon (Peg-IFN) limits viral DNA replication but rarely leads to FC (3). While prophylactic vaccines against HBV have proven safe and effective at preventing infection via induction of neutralizing antibody responses, they fail to induce T-cell responses capable of mediating FC when used therapeutically (4–6).

In CHB, the virus successfully evades immune control through a number of mechanisms, in part, due to the excessive production of subviral HBsAg particles. These are thought to suppress liver innate and adaptive immune responses by a variety of known and unknown mechanisms, resulting in impaired HBV-specific T-cell responses. To achieve immune control of viral replication, a multi-component approach is likely to be required to induce a reduction in HBsAg levels (7), followed by immune approach(es) including therapeutic vaccination to induce HBV-specific CD4^+^ and CD8^+^ T cells (8–10).

Components with different mechanisms of action are being combined in FC clinical trials that generally include an investigational HBsAg lowering agent [small interfering RNA (siRNA), antisense oligonucleotide (ASO), or HBsAg mAb] in combination with an immunomodulator and/or therapeutic vaccine candidate. Whereas clinical data regarding the efficacy of the siRNA plus therapeutic vaccination in a multi-component approach is currently unavailable as clinical trials evaluating such an approach are still ongoing, preclinical studies in the immune tolerant AAV-HBV mouse model of CHB provide preclinical proof of concept (11, 12). Michler and colleagues (13) demonstrated that, in AAV-HBV–transduced mice with moderate-to-low HBsAg levels (10^3^ IU/mL), sequential treatment with siRNA to reduce HBsAg followed by a therapeutic vaccination regimen with two priming doses of HBsAg and HBcAg protein and boosted with Modified Vaccinia Ankara (MVA)–expressing HBsAg and HBcAg (*TherVacB*) induced high-titer anti-HBsAg and anti-HBcAg neutralizing antibodies and HBV-specific T cells that resulted in sustained reduction of HBsAg, HBeAg, and HBV DNA below the limit of quantification. In contrast, therapeutic vaccination or siRNA treatment alone did not induce anti-HBV neutralizing antibodies nor HBV-specific T cells and failed to durably suppress HBsAg to undetectable levels (13).

Here, we extend these observations to a translational AAV-HBV mouse model setting with higher HBsAg levels 10^4^–10^5^ IU/mL, i.e., levels that resemble those observed in the majority of CHB patients on SOC. We demonstrate that therapeutic vaccination with a lipid nanoparticle (LNP)–formulated, self-amplifying RNA vaccine (i.e., replicon) alone was able to break immune tolerance in mice with low HBsAg titers (≤ 10^2^ IU/mL), inducing polyfunctional HBV-specific T cells, durable suppression of serum HBsAg below the limit of quantification, and elimination of HBsAg+ hepatocytes. In contrast, therapeutic vaccination of mice that initially had very high HBsAg titer (≥10^5^ IU/mL) that was brought low (10^2^ IU/mL) prior to vaccination by HBV siRNA pre-treatment was ineffective at reducing HBsAg and inducing HBV-specific T cells with evidence of exhaustion, defined as progressive loss of secreted antiviral cytokines Interleukin-2 (IL-2), tumour necrosis factor alpha (TNFα), and interferon gamma (IFNγ) and upregulation of immune-inhibitory surface molecules like PD1, similar in phenotype to T cells in the liver of CHB patients. We therefore screened immunomodulatory agents in this modified AAV-HBV mouse model that target T cells, B cells, or macrophages to understand the nature of immune suppressive pathways and identify which component(s), when combined with HBV therapeutic vaccination, may enhance the clearance of HBV-expressing cells. We report here that programmer death-ligand 1 (PDL1) blockade in combination with co-inhibitory pathways were largely ineffective at augmenting the efficacy of therapeutic vaccination; however, anti-PDL1 with stabilized cytokines IL-2 or IL-15 led to immune control of HBsAg in HBV replicon vaccinated animals.

## Materials and methods

### Animal studies

All studies were designed and performed in accordance with an Animal Use Protocol approved by an Institutional Animal Care and Use Committee and performed in Association for Assessment and Accreditation of Laboratory Animal Care (AAALAC)-accredited facility (Explora Biolabs, San Diego CA). Six to 8-week-old C57/BL6 mice (Jackson labs) were inoculated intravenous (i.v.) with indicated viral genome equivalents (v.g.e.) of rAAV8-1.3xHBV construct (serotype ayw; BrainVTA, China). High-titer infection (HBsAg at >10^4^ IU/mL) was generated using 5 × 10^10^ v.g.e., 5 × 10^9^ v.g.e. for mid titer (HBsAg at ~10^3^ IU/mL), and 5 × 10^8^ v.g.e. for low titer (HBsAg ~10^1^–10^2^ IU/mL). After 4 weeks, animals were randomized into treatment groups on the basis of the HBsAg level and treated as indicated. For siRNA treatment, animals were injected subcutaneous (s.c.) with GalNAc-conjugated HBV-siRNA (3 mpk). Two weeks later, animals were given a second siRNA dose and 3 µg of a tetracistronic replicon HBV therapeutic vaccine (Tetra-3) administered via bilateral intramuscular (i.m.) injection. Self-replicating tetracistronic vaccine was formulated in LNP or left unformulated. Control groups were inoculated with either saline or alpha 1 antitrypsin (AAT)-encoded negative control siRNA sequence.

The following antibodies targeting indicated murine immune pathways were purchased from BioXcell (Lebanon, New Hampshire) and 100 µg injected intraperitoneal (i.p.) weekly: anti-programmed death ligand-1 (PDL1), anti-cytotoxic T-lymphocyte-associated protein 4 (CTLA4), anti-lymphocyte-activation gene 3 (Lag3), anti-T cell immunoglobulin and mucin domain-containing protein 3 (Tim3), anti-IL-10R, anti-V-domain immunoglobulin suppressor of T cell activation (VISTA), anti-glucocorticoid-induced TNF receptor family-related protein (GITR), anti-cluster of differentiation 73 (CD73), anti-OX40 ligand (OX40L), anti-4-1BBL (CD173), anti–IL-2 (JES6), anti–IL-2 (S4B6), anti-CD27, anti-Interferon-alpha/beta Receptor (IFNAR), and isotypes Immunoglobulin G subclass 1 (IgG1) and IgG2a. One microgram of IL-2 (ThermoFisher) was complexed with 5 µg of anti–IL-2 antibody per animal. Five micrograms of IL-2–fragment crystalizable (Fc) and IL-21–Fc or 10 µg of Fms-like tyrosine kinase 3 ligand (FLT3L)-Fc was administered per dose (Adipogen, San Diego, CA). One microgram of IL-15 (R&D systems, Minneapolis, MN) and 5 µg of IL-15R-Fc per animal were mixed fresh for 20 min prior to dosing per animal. R848 (resiquimod, Sigma, St. Louis, MO) was dosed at 10 µg. To deplete CD4 or CD8 T cells, 100–200 µg of anti-CD8, anti-CD4, or isotype control IgG2a (BioXcell) were injected i.p. as indicated 1 day prior to each vaccination. To monitor infection 50 µL of serum was collected at indicated time points, every 1–2 weeks, for circulating HBsAg, HBeAg, HBV DNA, and anti-HBs antibody responses. At the end of studies, animals were euthanized, and liver and spleens were isolated and processed to single-cell suspensions for *ex vivo* enzyme-linked immunospot (ELISpot) and intracellular cytokine staining (ICS) assays to assess antigen-specific T-cell activity.

### Enzyme-linked immunosorbent assay (ELISA)

Levels of secreted HBV surface and core antigens in serum of mice were quantified using HBsAg Chemiluminescence Immunoassay (CLIA) and HBeAg CLIA (Ig Biotechnology, Burlingame, CA), respectively. The HbsAg assay range was 0.05–250 IU/mL performed at both 1:50 and 1:1,200 dilutions for a lower limit of quantification (LLOQ) of 2.5 IU/mL and upper limit of quantification (ULOQ) of 300,000 IU/mL. The HBeAg assay range was 0.1–200 Paul Erlich Institute Units (PEIU)/mL performed at both 1:50 and 1:1,200 dilutions for a LLOQ of 5 IU/mL and ULOQ 240,000 IU/mL. Anti-HBs and anti-HBe responses were quantified using anti-HBs and anti-HBe CLIA (Ig Biotechnology). The anti-HBs assay range was 5–1,000 mIU/ml performed at 1:10 dilution for a LLOQ of 50 mIU/mL and ULOQ of 10,000 mIU/mL. Assays were performed according to the manufacturer’s instructions, read on plate reader (Spectramax3), and analyzed using Prism Software. Alanine Aminotransferase (ALT) levels in serum were measured by ALT activity assay, with LLOQ of 0.2 U/mL (Sigma).

### qPCR

DNA was extracted from 50–100 µL of serum using a QIAamp 96 DNA Blood kit (QIAGEN, Redwood City, CA). HBV DNA was quantified using primers 5′-GTGTCTGCGGCGTTTTATCA and 5′-GACAAACGGGCAACATACCTT and probe 5′-FAM CCTCTKCATCCTGCTGCTATGCCTCATC-3′ Tamra and FastStart Universal probe master mix (Roche). Samples were run on QuantStudio 6 (Applied Biosystems, Foster City, CA) alongside plasmid DNA standards and normalized to copies/mL, with LLOQ of 200,000 copies/mL.

### IHC

Animals were perfused with 20 mL of cold Phosphate buffered saline (PBS), and liver lobes were placed in 10% formalin jars and processed by Histowhiz (New York, NY). Samples were paraffin embedded and stained for HBs and HBV Core proteins. Images were scanned and quantified. Percentage area positive was determined by 3,3-diaminobenzidine (DAB) tetrahydrochloride staining and graphed next to representative images.

### HBV peptides

Peptide pools of each HBV protein matching Tetra-3 replicon sequences (Core, Pol, PreS2.S, and PreS1 proteins) were designed in-house and custom synthesized as 15-mer peptides with 11–amino acid overlap (JPT Peptide Technologies, Germany). The large 94-kDa HBV Pol protein was split in the middle to yield two separate peptide pools. The peptide N- and C-termini that were acetylated and amidated, respectively, were of >80% purity as determined by mass spectrometry and contained endotoxin (<0.01 EU/µg). All peptide pools were lyophilized and resuspended in Dimethylsulfoxide (DMSO) at a concentration of 1 mg/mL of each peptide.

### ELISpot

For ELISpot analysis, 2 × 10^5^ splenocytes were added to IFNγ detection 96-well plates (Mabtech, Cincinnati, OH) in duplicate and stimulated with peptide pools spanning Core, Pol, PreS2.S, or PreS1. Following overnight incubation, plates were immunoblotted to quantitate IFNγ-positive spot-forming cells (SFCs) following the manufacturer’s protocol. Spots were counted using an automated CTL ImmunoSpot counter (Cellular technology Limited, Cleveland OH) and graphed as DMSO background-subtracted SFC per million splenocytes in Prism Software, with LLOQ of 5 spots/million cells.

### Flow cytometry

To isolate intrahepatic lymphocytes, animals were perfused through the heart with 20 mL of cold PBS. Livers were dissected, placed in a C tube (Miltenyi Biotec) containing 5 mL of complete media, and homogenized using the gentleMACS Octo Dissociator (Miltenyi Biotec). Homogenized liver tissue was poured through a 100-µm cell strainer and rinsed with 15 mL of media. Eight milliliters of Percoll (Cytiva) was added to each tube and centrifuged at 500 g for 20 min with no brake. Supernatant was aspirated, and red blood cells were lysed. Tubes were centrifuged at 300 g for 5 min at 4°C, and the remaining pellet was resuspended in media for downstream analysis.

For ICS analysis, single-cell suspensions were plated at 1 million cells per well and stimulated with Core, PreS1+PreS2.2, and Pol 1 + Pol 2 peptides (1 µg/mL) at 37°C. After 1 h, a cocktail containing Brefeldin A (eBioscience), Monensin (eBioscience), and CD107a (1D4B, BioLegend) was added, and cells were incubated for 5 more hours. Cells were stained for surface markers anti-CD3e (145-2C11, BD Biosciences), anti-CD4 (GK1.5, BD Biosciences), anti-CD8a (53-6.7, BD Biosciences), anti-CD279 (29F.1A12, BioLegend), anti-Ly-6C (HK1.4, BD BioLegend), anti-CD44 (IM7, BioLegend), anti-LAG3 (C9B7W, BioLegend), anti-CD25 (PC61, BioLegend), and anti-NK1.1 (PK136, BioLegend); fixed with Cytofix (BD biosciences); and permeabilized with 1× Perm/Wash (BD biosciences) and then intracellularly stained according to the manufacturer’s protocol with the following antibodies: anti-IFNγ (XMG1.2, BioLegend), anti–Granzyme A (GzA-3G8.5, ThermoFisher), anti–IL-2 (JES6-5H4, BioLegend), and anti-TNFα (MP6-XT22, BioLegend). After staining, cells were washed twice with 1× Perm/Wash buffer and resuspended in 150 μL of 1× FACS buffer. Data were acquired using BD Fortessa flow cytometer (Bennet Dickson) and analyzed in FlowJo software version 10 (TreeStar, Oregon). The frequencies of CD4+ or CD8+ T cells expressing CD107a and IFNγ were quantified, and the percentage of those peptide responding cells expressing TNFα, IL-2, PD1, LAG, Tim3, and CTLA4 was determined.

## Results

### HBV SMARRT therapeutic vaccine efficacy is influenced by serum HBsAg concentrations

**Figure 1:**
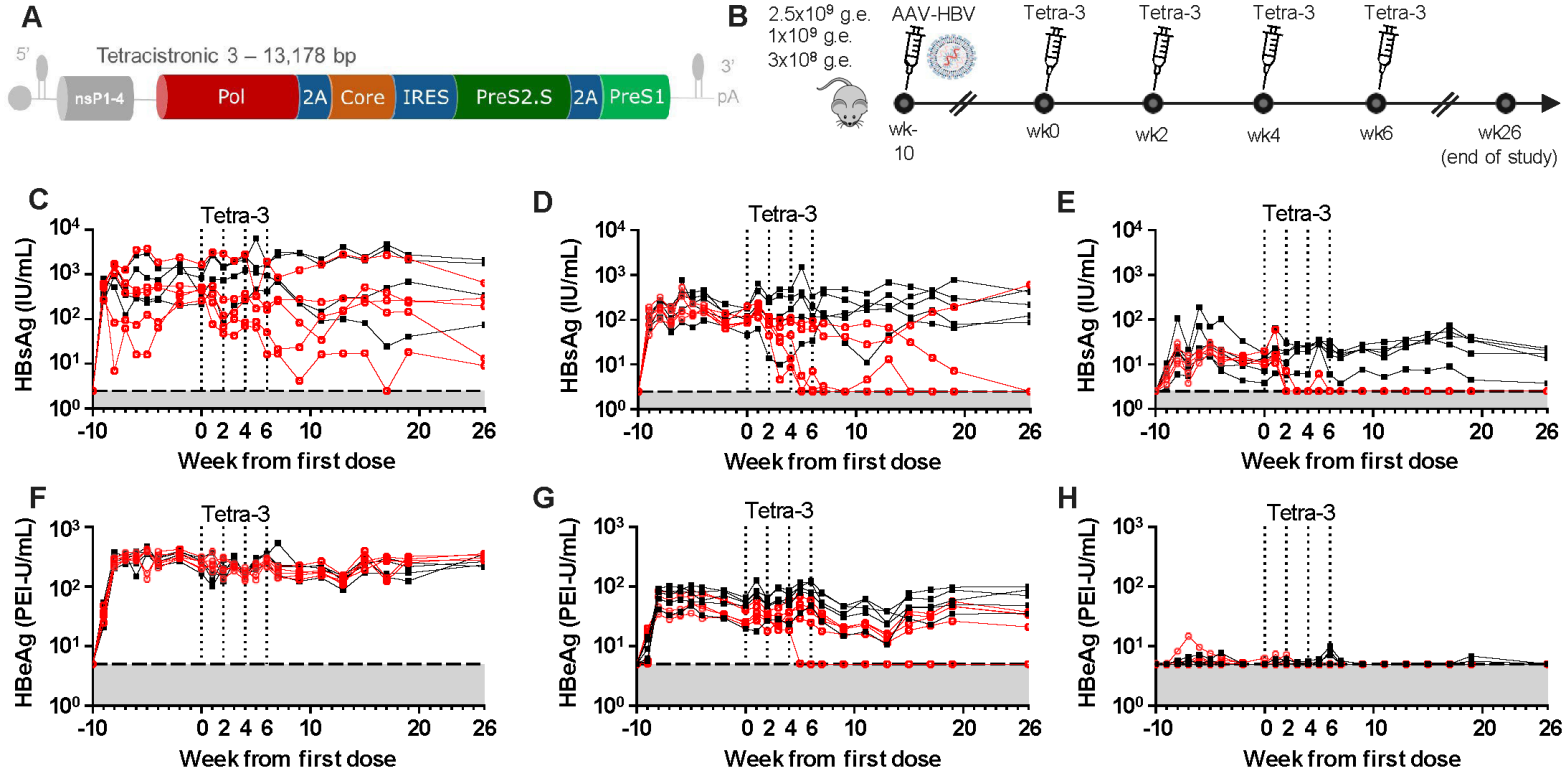
HBV SMARRT Therapeutic Vaccine efficacy is inversely affected by serum HBsAg concentrations. **(A)** Schematic of Tetra-3 HBV SMARRT self-replicating mRNA vaccine encoding four HBV proteins. **(B)** Dosing schematic of Tetra-3 efficacy study. Ten-week-old animals were infected with three different levels of AAV-HBV to establish chronic infection with high, medium, and low circulating HBsAg. High-dose [2.5 × 10^9^ viral genome equivalents (v.g.e.)], mid-dose (1 × 10^9^ v.g.e.), and low-dose (3 × 10^8^ v.g.e) inoculum. **(C–E)** HBsAg in serum of mice over time in High-titer **(C)**, Mid-titer **(D)**, and Low-titer **(E)** infection shown as individual mice, vehicle (black square), and Tetra-3 (red circle). Tetra-3 dosing indicated with dotted lines, four doses every 2 weeks, first dose labeled as Time 0. **(F–H)** HBeAg in serum over time as above. N = 5 per group shown as individual animals, limit of detection in gray.

The optimization of a novel LNP-formulated self-amplifying RNA (replicon) HBV therapeutic vaccine, based on the Synthetically Modified Alpha Replicon RNA Technology (SMARRT) vaccine platform, that expresses multiple HBV antigens will be described separately [Marro et al., manuscript in preparation (14)]. The SMARRT vaccine platform is a self-amplifying RNA derived from the genome of the TC−83 attenuated vaccine strain of the Venezuelan Equine Encephalitis Virus alphavirus, optimized to induce high-quality and high-magnitude antigen-specific CD4+ and CD8^+^ T cells (15). Here, we assessed the efficacy of a Tetra-3 HBV replicon therapeutic vaccine (expressing four major HBV proteins subsequently referred to as Tetra-3) (**Figure 1A**) to durably reduce HBV viral markers in the immune tolerant AAV-HBV–transduced mouse model of CHB. High serum levels of HBsAg, comprised primarily of excess HBV subviral particles, impair immune responses to HBV antigens *in vivo* including in the AAV-HBV–transduced murine model (13, 16, 17); hence, we similarly calibrated the efficacy of Tetra-3 in three different cohorts of AAV-HBV–transduced mice with serum HBsAg concentrations of 10^3^ IU/mL, 10^2^ IU/mL, and 10^1^ IU/mL. Mice were dosed intramuscularly with 10 µg of Tetra-3 formulated in LNPs every 2 weeks for a total of four doses (**Figure 1B**).

**Figure 2:**
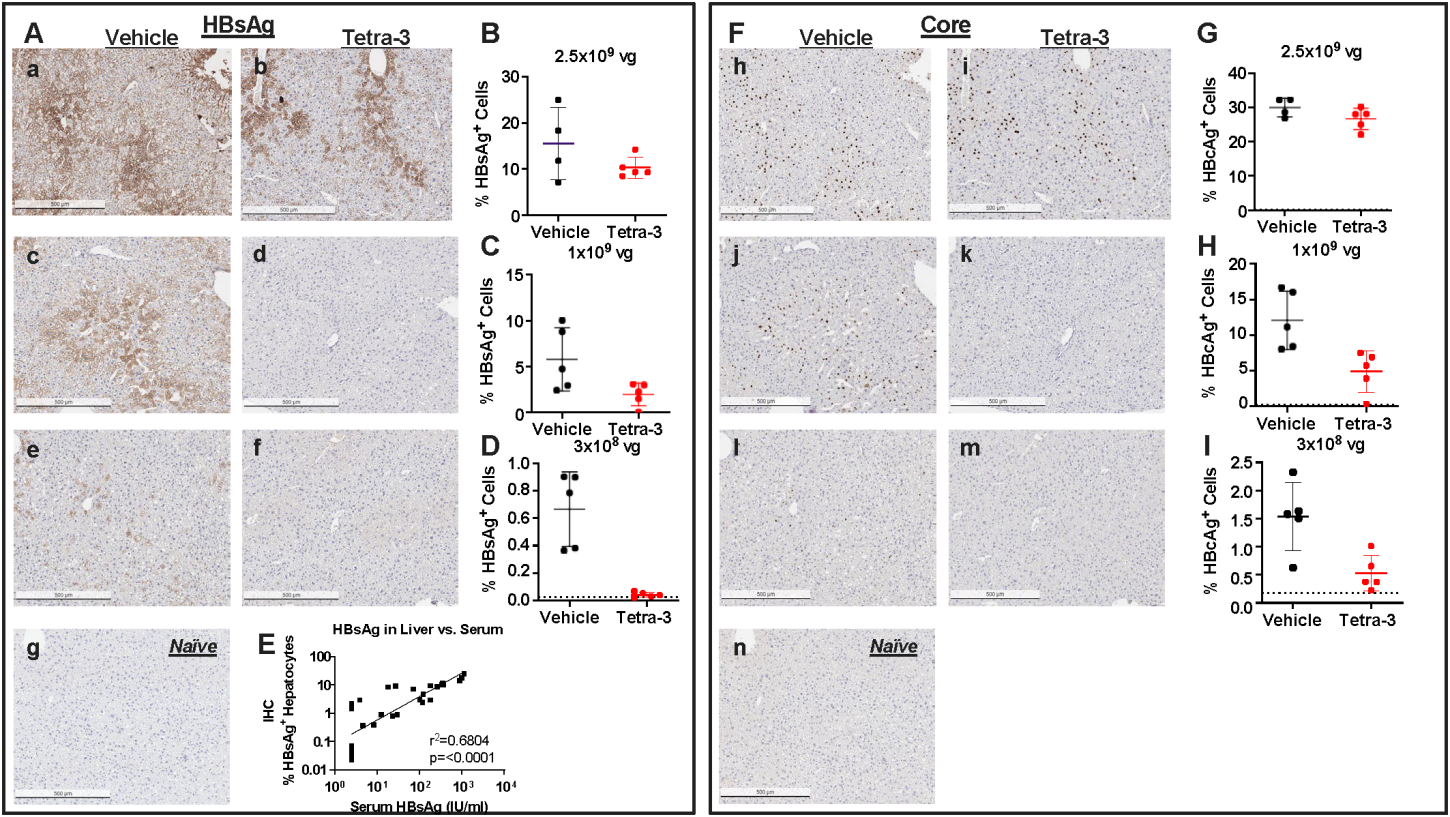
HBV therapeutic vaccine efficacy influenced by Liver HBsAg levels. **(A)** Histology and quantification of liver HBsAg at end of study week 26 post–first vaccine dose as in **Figure 1**. Representative sections of High-titer infection treated with vehicle (a) or Tetra-3 (b). **(B)** corresponding quantification as percentage of cells staining positive for HBsAg. **(C)** Mid-titer infection treated with vehicle (c) or Tetra-3 treated (d) with quantification **(C)**. **(D)** Low-titer infection treated with vehicle (e) or Tetra-3 treated (f) and quantification in **(D)**, compared to naïve mice with no infection (g). **(E)** Correlation of liver quantification with serum HBsAg levels for individual animals. **(F-I)** Representative histology for HBcAg in high-titer infection **(h, I, H)**, mid-titer infection (j-I), and low-titer infection (l, m, J) compared to naive animals in panel (n). Naive animal group represented by dotted line.

Tetra-3 efficacy was inversely associated with baseline serum (**Figures 1C–H**) and liver (**Figure 2**) HBsAg levels. Tetra-3 was ineffective at controlling posttreatment viremia in mice with 10^3^ IU/mL baseline HBsAg, as no mice achieved durably undetectable HBsAg levels and HBeAg levels or reductions in HBV DNA levels after receiving up to four HBV replicon vaccine doses every other week (**Figures 1C, F**, **Supplementary Figure 1**). However, among mice with baseline HBsAg (10^2^ IU/mL), two to four doses of Tetra-3 reduced serum HBsAg concentrations to undetectable in three of the five mice, and HBV DNA levels were on average 1-log_10_ lower compared to those in untreated mice (**Figures 1D, G**). Furthermore, of these three HBsAg mice (10^2^ IU/mL) that had serum HBsAg reduced to undetectable, one mouse also had serum HBeAg reduced to undetectable (**Figure 1F**). Among mice with baseline HBsAg (10^1^ IU/mL), a single dose of Tetra-3 was sufficient to reduce serum HBsAg to undetectable in all mice (five of five) (**Figures 1E, H**). Efficacy of Tetra-3 in mice with baseline HBsAg (10^1^ IU/mL) as measured through HBeAg and HBV DNA markers was unclear because HBeAg and HBV DNA in untreated mice with baseline HBsAg (10^1^ IU/mL) were below the LLOQ (**Figure 1H**, **Supplementary Figure 1**). Notably, control of serum HBsAg in Tetra-3–treated mice was maintained until the end of study, which was 20 weeks after the final HBV replicon dose.

The profound posttreatment reductions of viremic markers were not only noted in serum samples but were also confirmed by immunohistochemistry staining of liver tissue isolated from the different cohorts of mice (**Figure 2**). Serum HBsAg and the percent of hepatocytes positive for HBsAg were highly and significantly correlated (r2 = 0.6804, P < 0.0001) (**Figure 2**). Tetra-3 dosed four times every other week significantly reduced the percent of HBsAg+ hepatocytes and HBcAg+ hepatocytes by 93.6% (P = 0.0009) and 65.5% (P = 0.0107), respectively, in mice with baseline serum HBsAg at 10^1^ IU/mL; 66.1% (P = 0.0483) and 60% (P = 0.0124), respectively, in mice with baseline serum HBsAg at 10^2^ IU/mL but did not affect the percent of hepatocytes positive for HBsAg (33.5% reduction, P = 0.1391) of HBcAg (11.1% reduction, P = 0.1934) in mice with serum HBsAg at 10^3^ IU/mL.

### Tetra-3 efficacy is mediated by HBV-specific adaptive immune responses

To determine if the efficacy of Tetra-3 was associated with the induction of HBV-specific or non-specific immune responses induced by LNP-formulated replicon RNA vaccine, we compared the efficacy of both LNP-formulated and unformulated Tetra-3 with that of the firefly luciferase control replicon in AAV-HBV–transduced mice with baseline levels of serum HBsAg (10^2^ IU/mL; **Supplementary Figure 2**). In this cohort of mice, only the groups treated with Tetra-3 had subsequent reductions in serum HBsAg. LNP-formulated Tetra-3 treatment reduced HBsAg to undetectable levels in three of the five mice after one to two doses, and unformulated Tetra-3 reduced serum HBsAg to undetectable in two of the five mice but required three doses. In contrast, immunization with LNP-formulated firefly luciferase control replicon had no effect on serum HBsAg, suggesting that Tetra-3 reduces HBsAg in the AAV-HBV mouse model by HBV-specific adaptive immunity.

Analysis of HBV-specific, IFNγ^+^ splenic T-cell responses by ELISpot 5 weeks after the last Tetra-3 dose revealed significantly higher HBV-specific T-cell responses in mice that were treated with LNP-formulated Tetra-3 (mean ± SD; 1,616 ± 348 spot forming units (SFU)/10^6^ cells) compared to mice treated with unformulated Tetra-3 (475 ± 181 SFU/10^6^ cells; P = 0.0002), which is consistent with the greater efficacy of LNP-formulated, compared to unformulated, Tetra-3 (**Supplementary Figure 2I**). Neither LNP-formulated Tetra-3 nor unformulated Tetra-3 induced detectable anti-HBsAg or anti-HBeAg antibodies (**Supplementary Figures 2J, K**). As expected, the firefly luciferase replicon vaccine did not induce HBV-specific T-cell responses above that seen in vehicle-treated mice (102 ± 77 SFU/10^6^ cells vs. 62 ± 27 SFU/10^6^ cells, respectively; P = 0.3084) nor detectable anti-HBsAg or anti-HBeAg antibodies.

**Figure 3:**
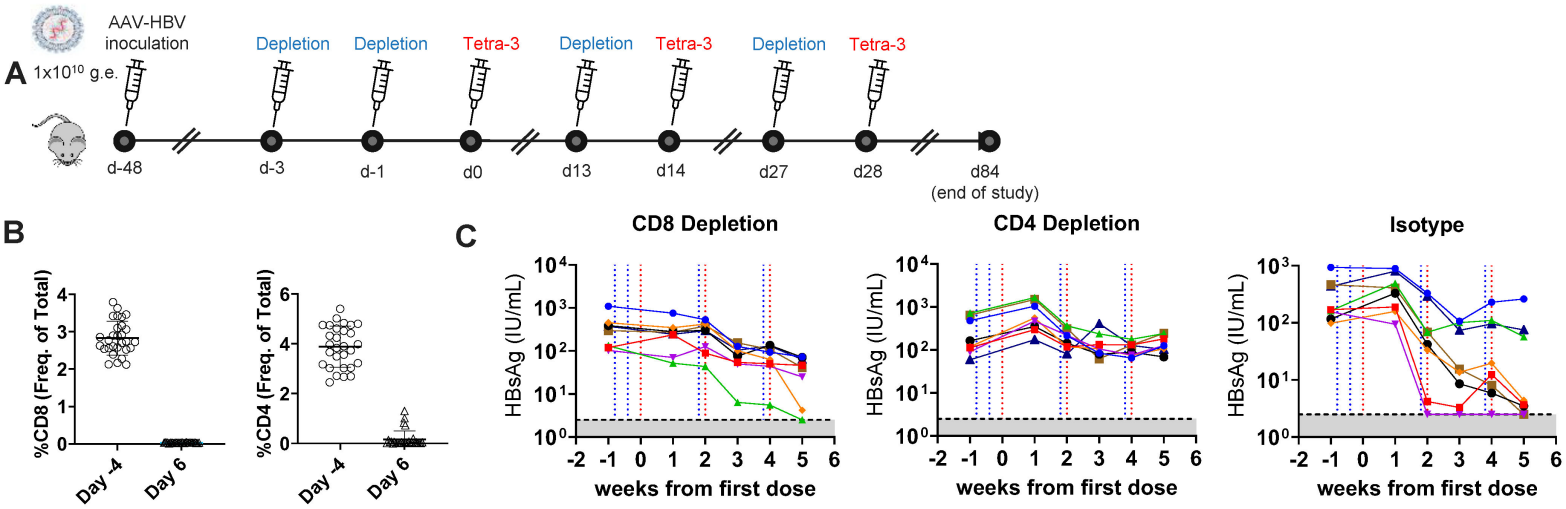
T cell–dependent control of HBsAg after therapeutic vaccination. **(A)** Schematic of T cell depletion. Mice were inoculated with AAV-HBV to establish mid-titer infection with HBsAg 10^2^–10^3^ IU/mL for ~7 weeks. **(B)** Frequency of total CD8 and CD4 T cells in blood by flow cytometry, 4 days before and 6 days after T cell depletion to confirm treatment. **(C)** Animals received 100 µg of anti-CD4, CD8, or isotype control antibody i.p. (blue dotted line) on days −3 and −1 prior to first Tetra-3 therapeutic vaccination mice i.m. (red dotted line) and 1 day prior to each subsequent vaccination, and the levels of HBsAg were monitored over time, with first Tetra-3 dose displayed as Time 0. N = 8 mice per group shown as individual animals, limit of detection in gray.

To further investigate the contribution of adaptive HBV-specific immunity to the efficacy of the Tetra-3, we assessed efficacy of Tetra-3 in AAV-HBV–inoculated mice with baseline HBsAg levels (10^2^ IU/mL) that were depleted of CD8 and CD4 T cells prior to vaccination. CD8 and CD4 T cells were depleted by intraperitoneal administration of 100 µg of anti-CD8 and anti-CD4 mAbs 3 days and 1 day prior to the Tetra-3 priming dose, and depletion was repeated 1 day prior to each subsequent replicon Tetra-3 dose on day 14 and day 28 (**Figure 3A**). Anti-CD8 and anti-CD4 mAbs depleted peripheral CD8 and CD4 T cells in this cohort of mice almost completely, except in two mice that retained 1% of CD8 T cells (**Figures 3A, B**), and we confirmed such treatment similarly depletes CD8 and CD4 T cells in the liver and spleen in a separate cohort of AAV-HBV–transduced mice (**Supplementary Figure 3**). Depletion of either CD8 T cells or CD4 T cells reduced or completely abrogated Tetra-3 efficacy, respectively, with only two of the eight mice in the CD8 T-cell–depleted group and none (zero of eight) of the CD4 T-cell–depleted mice achieving undetectable serum HBsAg levels, compared to five of the eight isotype control mAb and Tetra-3–treated mice that showed posttreatment undetectable serum HBsAg levels (**Figure 3C**). Two of the eight CD8-depleted animals that did control HBsAg also had residual CD8 T cells in blood, suggesting incomplete depletion (data not shown). Taken together, these data indicate that Tetra-3 HBV replicon vaccine induces high-magnitude HBV-specific T cells and is effective at reducing serum HBsAg in AAV-HBV–infected mice with serum HBsAg concentrations ≤ 10^2^ IU/mL in the absence of high-titer anti-HBsAg antibodies, suggesting a CD4 and CD8 T-cell–dependent mechanism.

### Peak, not pre-treatment, serum HBsAg concentrations limit replicon efficacy and immune responses

**Figure 4:**
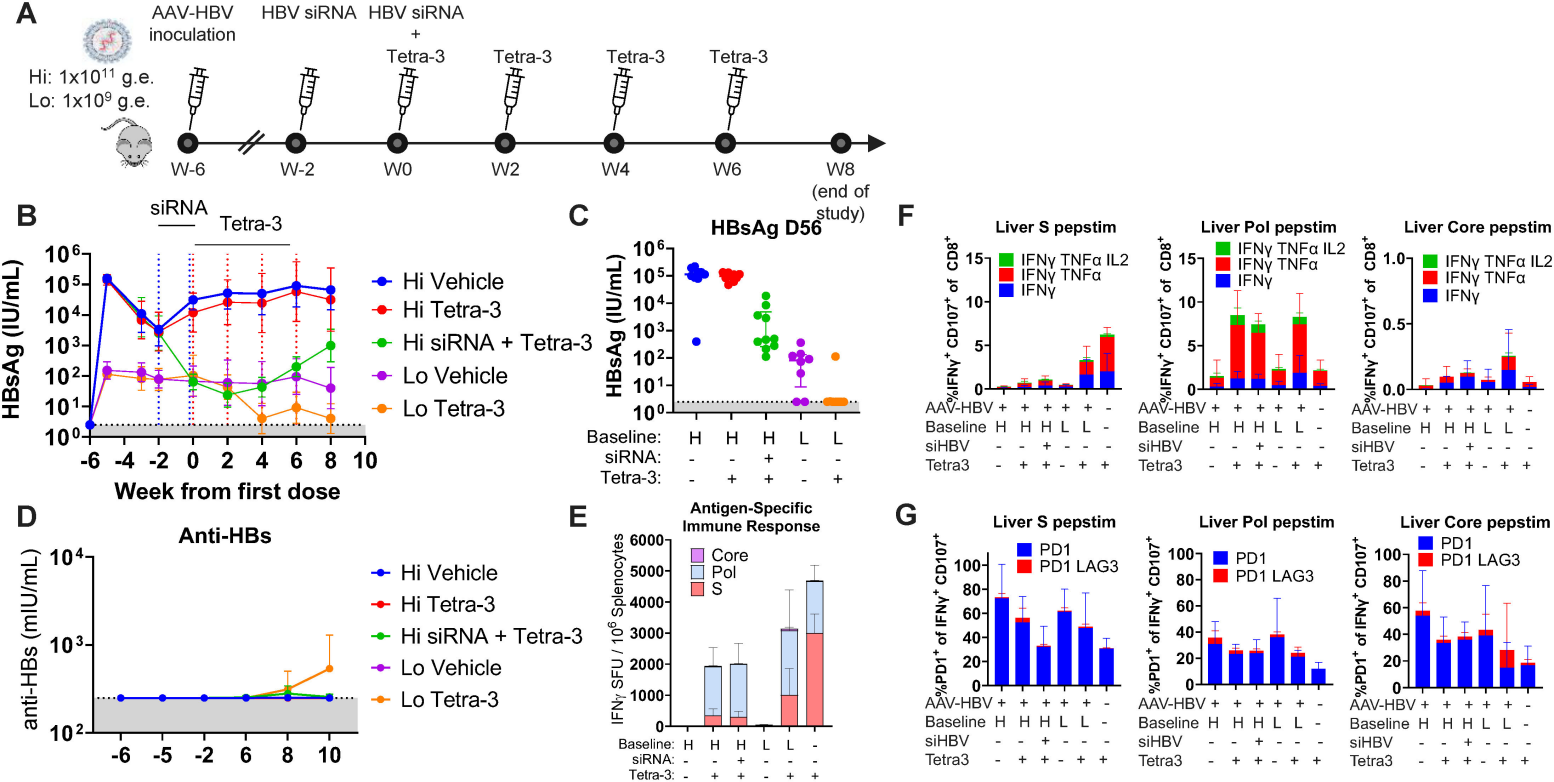
High-peak HBsAg reduces efficacy of therapeutic vaccination. **(A)** Schematic of experiment. Animals were inoculated with 1 × 10^11^ or 1 × 10^9^ v.g.e. of AAV-HBV to establish high and low levels of serum HBsAg, respectively (week −6). After 4 weeks of infection, animals indicated as high-titer animals were dosed with 100 µg of HBV targeting siRNA s.c. at week −2 and again 2 weeks later (week 0) alongside the first of 4 Tetra-3 therapeutic vaccinations i.m. in both high-and low-HBsAg cohorts. **(B)** HBsAg level over time, with week 0 set to the first Tetra-3 dose. **(C)** HBsAg levels of individual mice from **(B)** at week 8. **(D)** Anti-HBs antibodies in serum over time. **(E)** At week 8, the spleens were harvested and IFNγ responding T cells measured by ELISpot for HBV proteins, Pol, PreS1, and PreS2 pooled (S) and Core, normalized per million splenocytes. **(F)** Livers analyzed for IFNγ, TNF, and IL-2 by intracellular flow cytometry and graphed as percentage of IFNγ responding cells to indicated peptide pools producing IFNγ only (blue) or co-producing TNFα (red) and IL-2 (green). **(G)** Percentage of IFNγ responding CD8 T cells in liver expressing PD1 (blue) or PD1 and LAG3 (red). N = 8–10 per group shown as geometric mean ± 95% CI or individual mice for HBsAg and mean ± standard deviation for ELISpot and flow cytometry. Limit of detection in gray.

The HBV replicon vaccine is effective at reducing HBsAg in male AAV-HBV mice that have baseline serum HBsAg concentrations ≤ 10^2^ IU/mL but ineffective in mice with HBsAg ≥ 10^3^ IU/mL, which is consistent with an HBV therapeutic vaccination effect noted by others in a similar model (13). Clinically, the longitudinal serum viral profile in HBV-infected individuals may be more variable with high early transient levels that are not uncommon in the progression from acute to chronic infection or among individuals treated with HBsAg lowering agents currently in clinical trials. To adapt the AAV-HBV–transduced murine model to better reflect the course of CHB infection observed clinically, we assessed the HBV replicon therapeutic vaccine in mice wherein initially high baseline HBsAg levels were knocked down with siRNA prior to replicon dosing. Three groups of mice were transduced with AAV-HBV to yield stable serum HBsAg concentrations of 10^5^ IU/mL (High), and two groups of mice were transduced with AAV-HBV to yield serum HBsAg concentrations of 10^2^ IU/mL (Low). Four weeks post-transduction, one of the High mice groups were dosed with HBV-specific siRNA (referred to as the Hi-to-Lo group) to reduce HBsAg within 2 weeks to levels comparable to Low mice (10^2^ IU/mL), at which time all groups (except High and Low untreated control groups) were dosed with Tetra-3; the Hi-to-Lo group also received a second dose of HBV siRNA. All groups received three additional doses of Tetra-3 every other week (**Figure 4A**).

Consistent with the previous efficacy study, Tetra-3 reduced HBsAg to undetectable levels in Low HBsAg mice and was not effective in suppressing viremic markers in High HBsAg mice, with seven of eight Low mice and 0 of the 10 High mice achieving undetectable HBsAg at end of study (**Figures 4B, C**). Notably, among Hi-to-Lo mice, a full course of Tetra-3 dosing was ineffective with 0 of the 10 mice achieving undetectable HBsAg. Thus, the efficacy of therapeutic vaccination with Tetra-3 was influenced by historical peak serum HBsAg concentrations rather than serum HBsAg concentrations immediately prior to vaccination. Given the observation from our model that HBV viral markers may be controlled with vaccination of animals below a baseline/never-exceeded threshold of serum HBsAg, we proceeded to examine the HBV-specific immune responses. Anti-HBsAg antibodies were detected only in Low mice that were treated with Tetra-3, becoming detectable at week 8, which was 2 weeks after the second vaccine dose (**Figure 4D**). No anti-HBsAg antibodies were detected in High or Hi-to-Lo mice treated with Tetra-3 or in untreated animals (**Figure 4D**).

HBV-specific T-cell responses were evaluated *ex vivo* 2 weeks after the last Tetra-3 dose by ELISpot and ICS on single-cell suspensions of the spleen and liver after peptide stimulation with overlapping pools of 15-mer peptides to each HBV viral protein: Pol, HBsAg, and Core. Splenocytes of Tetra-3–treated animals showed increased Pol-specific IFNγ ELISpot responses across groups compared to vehicle-treated animals regardless of HBsAg titer (**Figure 4E**). However, HBsAg-specific responses were significantly reduced in both High-titer (354 ± 207 SFU/10^6^ cells) and in Hi-to-Lo–titer (302 ± 183 SFU/10^6^ cells) animals compared to that in Low-titer (1,016 ± 848 SFU/10^6^ cells) or uninfected animals (2,998 ± 617 SFU/10^6^ cells), which mounted robust HBsAg-specific responses. Similar trends were observed with HBV core-specific T-cell responses but were lower in overall magnitude relative to the HBsAg-specific T-cell responses (**Figure 4E**).

ICS on both isolated spleen and liver cells indicated that, in Low-titer mice, Tetra-3 induced IFNγ-producing polyfunctional CD8 T-cell responses, comparable to uninfected animals in response to *ex vivo* HBV peptide stimulation (**Figure 4F**). However, in High-titer and Hi-to-Lo–titer mice, Tetra-3–induced liver HBsAg- and core-specific CD8 T cells had reduced IFNγ response as well as reduced TNFα and IL-2 polyfunctionality compared to Tetra-3–induced CD8 T cells in Low-titer mice or naïve mice (**Figure 4F**). Again, Pol-specific CD8 T-cell responses were similarly increased and functional in the liver and spleen of Tetra-3–treated groups over non-vaccinated groups, producing both IFNγ and TNFα (**Figures 4E, F**, **Supplementary Figure 4**). These results suggest Tetra-3–induced HBsAg- and Core-specific T-cell responses were sensitive to HBsAg titer, due to physical deletion and/or functional exhaustion, whereas Pol specific responses were not.

To further characterize potential T-cell exhaustion in chronic AAV-HBV–transduced mice, we examined PD1 expression alongside other known co-inhibitory markers LAG3, TIM3, and CTLA4. We found AAV-HBV–transduced animals had increased PD1 expression, particularly in untreated High-titer mice, where, on average, 70% of HBsAg-specific, IFNγ^+^ liver-resident CD8 T cells expressed PD1 (**Figure 4G**). LAG3 was detected in only >10% of PD1-positive, IFNγ^+^-producing cells, and CTLA4 and TIM3 expression was not detected. In contrast, High-titer animals treated with Tetra-3 or siRNA had reduced liver PD1 expression on HBsAg-specific IFNγ^+^ cells, suggesting lower antigen exposure similar to that in Low-titer mice, although the PD1 expression level remained slightly above naïve mice (**Figure 4G**). Indeed, liver T-cell PD1 expression was higher on HBsAg- and core-specific IFNγ+ cells relative to Pol-specific IFNγ+ cells, with less than 50% of Pol-specific T cells expressing PD1. Interestingly, PD1 levels on HBsAg-specific (r2 = 0.1594, P = 0.0118) and Core-specific (r2 = 0.1112, P = 0.0380), but not Pol-specific (r2 = 0.0240, P = 0.3462) liver-resident IFNγ+ CD8+ T cells, correlated with serum HBsAg level (**Supplementary Figure 4C**). PD1 expression on HBsAg-specific (r2 = 0.004, P = 0.6895) and Core-specific (r2 < 0.0001, P = 0.9576) IFNγ+ splenic CD8 T cells also did not correlate with serum HBsAg levels (**Supplementary Figure 4D**). Importantly, high historical peak HBsAg titer during the first 4 weeks following AAV-HBV transduction was sufficient to induce T-cell tolerance to HBsAg and Core epitopes, which persisted even once HBsAg levels are reduced by HBV siRNA.

### Identifying T-cell exhaustion pathways in Hi-to-Lo AAV-HBV model

The different results following (short-term) siRNA-induced HBsAg reduction and therapeutic vaccination (Hi-to-Lo model), relative to vaccinated mice that only had low-titer HBsAg, provided for a robust pharmacological model to identify specific interventions that in combination with siRNA HBsAg knockdown and therapeutic vaccination may lead to sustained HBV immunological control. We first focused on examining the functional requirements for T-cell exhaustion in our High-to-Low–titer model, wherein we performed *in vivo* blockade of various pathways in addition to anti-PDL1 in two large cohorts of animals (male and female cohorts).

**Figure 5:**
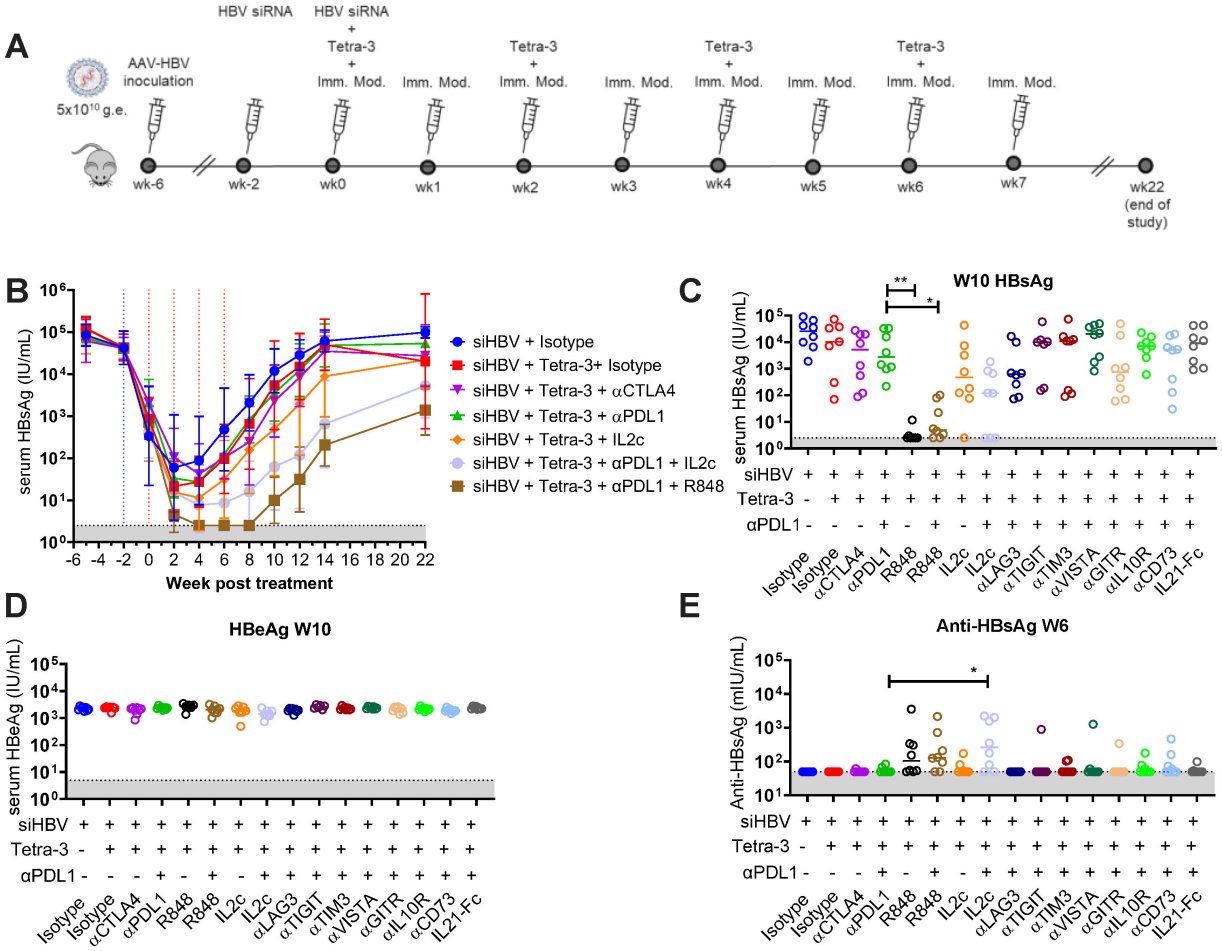
Exhaustion pathway screen for restoration of anti-HBV immune response in high-titer–infected male mice. **(A)** Schematic of experiment. Mice infected to high titer (5 × 10^10^ v.g.) of AAV-HBV intravenously on week −6. All groups were dosed with 100 μg of HBV-siRNA sub-cutaneous at weeks −2 and 0. At weeks 0, 2, 4, and 6, mice were given 3 μg of Tetra-3 therapeutic vaccine intramuscularly. At weeks 0, 2, 3, 4, 5, and 6, mice were given 100 µg of α-PDL1 and/or indicated immuno-modulator intraperitoneally, select groups shown. **(B)** Serum HBsAg levels over time. **(C–E)** HBsAg levels **(C)**, HBeAg **(D)**, and anti-HBs antibodies **(E)** in serum of individual mice from all treatment groups shown at week 10 postvaccination. N = 8 per group plotted as geometric mean and error with 95% CI or individual mice. Limit of detection in gray. Kruskal–Wallis ANOVA compared to siRNA + Tetra-3 + isotype group as control, *p < 0.05, **p < 0.0001.

PD1/PDL1 is a main pathway inducing T-cell tolerance in chronic infections in mice and HBV-specific T cells *ex vivo* in patients (18). Co-inhibition of additional inhibitory pathways often synergizes with PDL1 blockade to restore T-cell exhaustion but show minimal efficacy as single agents (19). To this end, we co-dosed Tetra-3 with anti-PDL1 (3 mg/kg), alone or in combination, with commercially available non-depleting mAbs blocking co-inhibitory molecules CTLA4, LAG3, TIGIT, TIM3, IL-10R, VISTA, or CD73, in the Hi-to-Lo model after HBsAg levels were reduced by siRNA for 2 weeks (**Figure 5A**). Anti-PDL1 or anti-CTLA4 alone did not augment control of HBsAg over the course of 4 biweekly vaccines doses and weekly target-specific antibody dosing as indicated (**Figures 5B, C**). Additional blocking of a second exhaustion pathway alongside anti-PDL1 also did not reduce HBsAg (**Figure 5C**), consistent with lack of strong co-inhibitory molecule expression in our AAV-HBV mouse model. Similarly, addition of agonist mAb against GITR or recombinant IL-21–Fc also did not reduce HBsAg or HBeAg (**Figures 5C, D**).

In contrast, co-dosing Tetra-3 with IL-2:S4B6 complex (1 µg of IL-2 complexed with 5 µg of anti–IL-2 clone S4B6) in combination with anti-PDL1 (3 mg/kg) significantly reduced HBsAg compared to co-dosing Tetra-3 with either an isotype control antibody (P = 0.0124) or with anti-PDL1 (P = 0.0362), comparable to co-dosing Tetra-3 with TLR7/8 ligand R848 (P = 0.1803), which has been demonstrated previously as monotherapy to reduce HBsAg in high-titer AAV-HBV–transduced mice (20) (**Figures 5A–C**).

Tetra-3–treated mice co-dosed with anti-PDL1 plus IL-2:S4B6 complex had significantly improved cellular and humoral responses compared to mice treated with only anti-PDL1 during Tetra-3 dosing. Analysis of peripheral T cells and serum isolated at week 12 indicated that Tetra-3–treated mice co-dosed with PDL1 plus IL-2:S4B6 complex had significantly higher absolute CD8 T-cell counts (P < 0.0001), significantly higher frequency of effector memory (defined as CD44+/CD62L+) CD8 (P = 0.0192) and CD4 (P < 0.0001) T cells, and significantly higher serum anti-HBsAg antibody levels (P = 0.0407) compared to Tetra-3–treated mice co-dosed with anti-PDL1 (**Figure 5E**, **Supplementary Figures 5A–C**).

**Figure 6:**
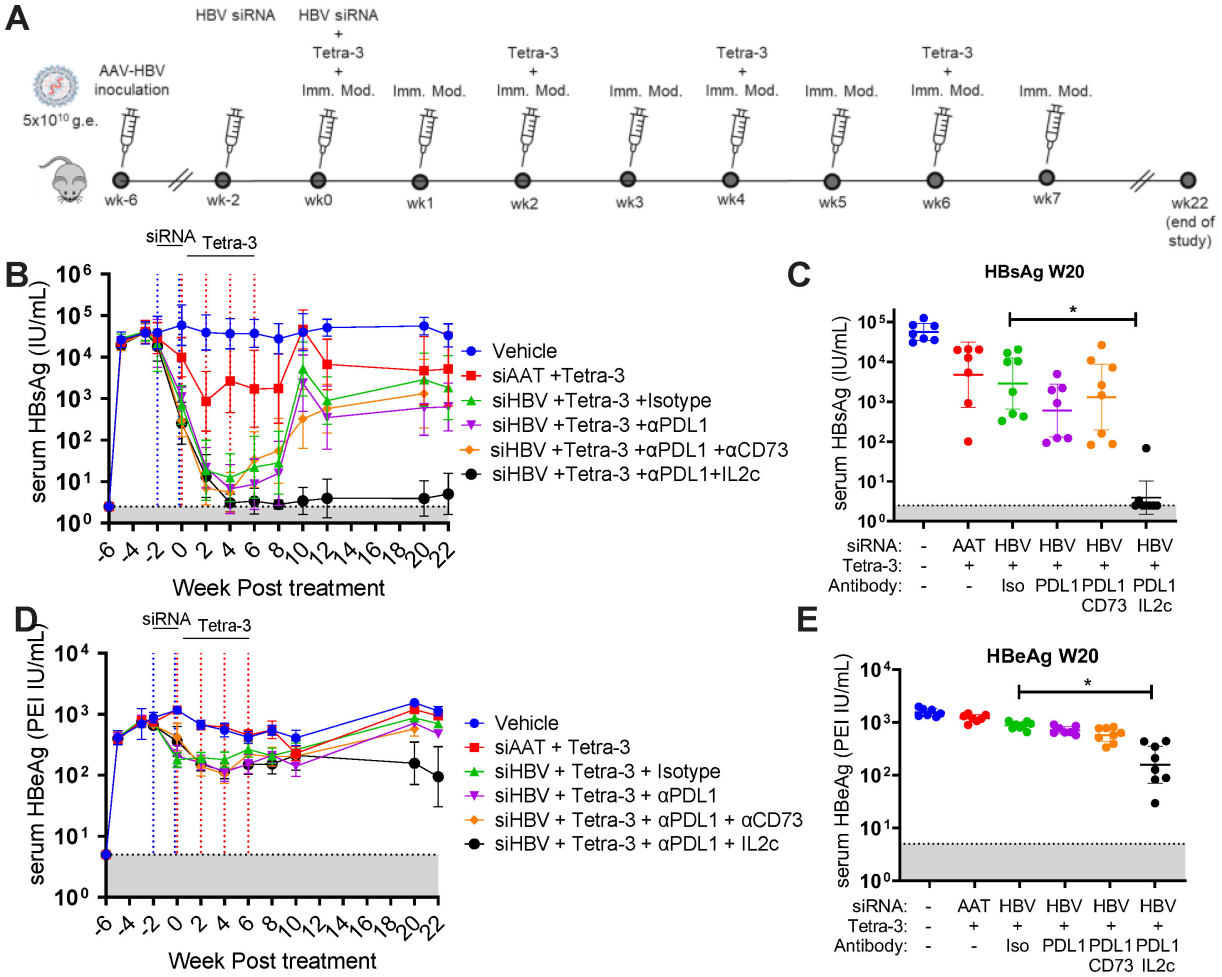
Exhaustion pathway screen for restoration of anti-HBV immune response in high-titer–infected female mice. **(A)** Schematic of experiment. Mice infected to high titer (5 × 10^10^ vg) of AAV-HBV intravenously on week −6. Mice received 100 μg of AAT-control siRNA or HBV-siRNA sub-cutaneous at weeks −2 and 0 as indicated (blue dotted lines). At weeks 0, 2, 4, and 6, mice were given 3 μg of Tetra-3 therapeutic vaccine intramuscularly (red dotted lines). At weeks 0, 2, 3, 4, 5, and 6, mice were given 100 µg of α-PDL1 and/or indicated immuno-modulator intraperitoneally. **(B)** Serum HBsAg levels over time. **(C)** Serum HBsAg levels of individual mice at week 20 postvaccination. **(D)** Serum HBeAg over time. **(E)** Serum HBeAg levels of individual mice at week 20. N = 7–8 mice per group shown as geometric mean ± 95% CI or individual mice. Kruskal–Wallis ANOVA compared to siRNA + Tetra-3 + Isotype group as control, *p < 0.05.

We confirmed the efficacy of IL-2:S4B6 complex in a cohort of female mice. Consistent with results in male mice, co-dosing Tetra-3 with a combination of IL-2:S4B6 complex plus anti-PDL1 following siRNA treatment led to prolonged suppression of HBsAg for >3 months compared to the same regimen without IL-2 (siRNA and Tetra-3 plus anti-PDL1) (**Figures 6A–C**). Female Tetra-3–treated mice co-dosed with anti-PDL1 plus IL-2:S4B6 complex increased control of HBsAg and a concomitant ~1-log reduction in HBeAg, suggesting improved treatment-induced viral control in female mice compared to that in male mice (**Figures 6D, E**). Overall female mice validated the IL-2:S4B6 complex approach and showed more pronounced viral control compared male mice with the same treatment regimen.

### IL-15 co-stimulation augments HBV replicon vaccine efficacy in Hi-to-Lo AAV-HBV model

**Figure 7:**
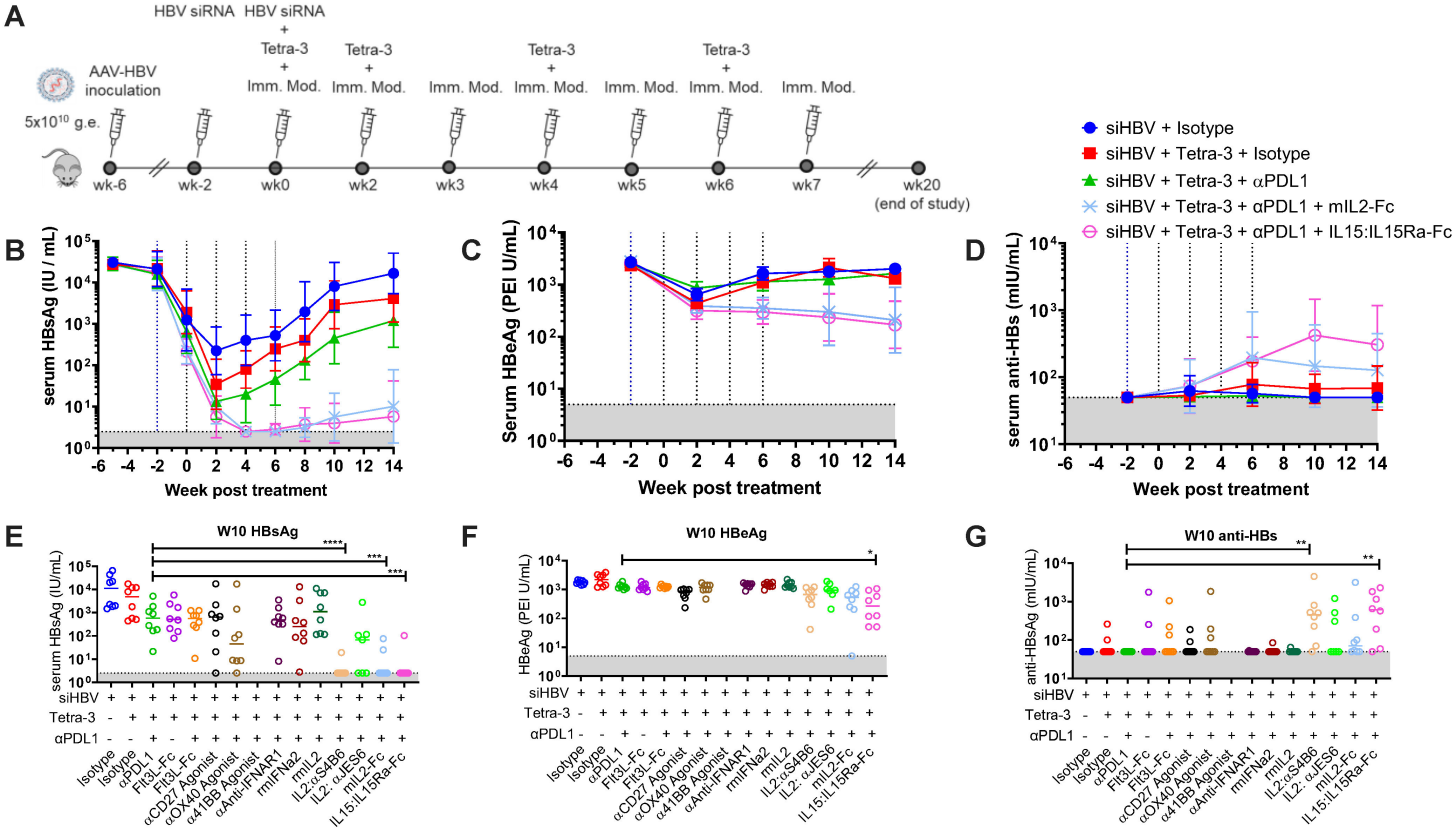
Co-stimulation pathway screen for restoration of anti-HBV immune response in high-titer–infected female mice. **(A)** Schematic of experiment. Mice infected to high titer (5 × 10^10^ vg) of AAV-HBV intravenously on week −6. Mice were given 100 μg of AAT-control siRNA or HBV-siRNA sub-cutaneous at weeks −2 and 0 as indicated (blue dotted lines). At weeks 0, 2, 4, and 6, mice were dosed with 3 μg of Tetra-3 therapeutic vaccine intramuscularly (red dotted lines). At weeks 0, 2, 3, 4, 5, and 6, mice were dosed with 100 µg of α-PDL1 and/or indicated immuno-modulator intraperitoneally. **(B–D)** serum HBsAg levels **(B)**, HBeAg level **(C)**, and anti-HBs antibodies **(D)** over time. **(E–G)** HBsAg **(E)**, HBeAg **(F)**, and anti-HBs **(G)** for individual animals for all groups at week 10 posttreatment. N = 7–8 mice per group shown as geometric mean ± 95% CI or individual mice. Kruskal–Wallis ANOVA compared to siRNA + Tetra-3 + isotype group as control, *p < 0.05, **p < 0.0001.

To further examine the role of IL-2 complex in augmenting the efficacy of replicon therapeutic vaccine and to assess the efficacy of additional T-cell (co)-stimulatory pathways, we performed another screen of targets in female mice using a similar study design as described above but we used different forms of recombinant IL-2 (IL-2 JES6 complex, IL-2, and IL-2–Fc), and we substituted antagonist antibodies to target select co-inhibitory receptors with agonistic antibodies or recombinant cytokines (IL-15) to T-cell co-stimulatory molecules (**Figure 7A**). Co-dosing Tetra-3 with anti-CD27, anti-OX40, anti-41BB agonists, anti-IFNAR blocking antibody, FLT3 ligand-Fc, or recombinant type I Interferon did not improve the efficacy of the combination of siRNA followed by Tetra-3 plus anti-PDL1. Anti-41BB treatment led to pathology, elevated ALT after treatment, and early removal of animals and was the only overt pathology observed across all studies (**Supplementary Figures 6A–C**).

In contrast, co-dosing Tetra-3 with specific IL-2 or IL-15 cytokine treatments led to significantly decreased serum HBsAg and HBeAg, compared to Tetra-3 co-dosed with isotype control antibody or anti-PDL1 alone (**Figures 7B, C, E, F**). Recombinant mouse IL-2 and IL-2 complexed with anti–IL-2 clone JES6, which targets CD25^high^ cells such as Tregs, did not augment the efficacy of Tetra-3 co-dosed with anti-PDL1. However, IL-2–Fc, which has an extended half-life compared to IL-2, was similarly efficacious as IL-2:S4B6 complex. In addition, the use of IL-15 complexed with IL-15Ra–Fc extends the half-life of IL-15 and allows for direct signaling on T-cell and NK cells expressing the same CD122 and CD132 shared subunits of IL-2 family of cytokines. Both IL-2 and IL-15 induced anti-HBs antibody response in treated animals 2 weeks post-boost to levels above 10^2^ mIU/mL (**Figures 7D, G**). Overall, these results suggest that increased T-cell proliferation alongside anti-PDL1 blockade, but neither treatment effect alone, can improve HBV therapeutic vaccine efficacy in the AAV-HBV chronic infection model. The role and phenotype of individual cell types, CD4 vs. CD8 T cells, or NK cells remains to be elucidated.

## Discussion

In CHB patients and the AAV-HBV mouse model of CHB, high levels of HBsAg have been described as one of the factors leading to immune tolerance to HBV antigens and impaired immune responses to HBV therapeutic vaccination and immune control of CHB infection. Here, we demonstrate in the AAV-HBV mouse model that historical peak serum HBsAg concentrations, not serum HBsAg concentrations immediately prior to therapeutic vaccination, influences the efficacy of therapeutic vaccination and that efficacy in clinically-relevant high HBsAg titer mice could be achieved by co-dosing Tetra-3 HBV replicon therapeutic vaccine with T-cell co-stimulators such as IL-2-Fc, IL-2:S4B6 complex or IL-15–IL-15Ra–Fc complex in combination with T-cell exhaustion antagonist, anti-PDL1 antibody.

The AAV-HBV mouse model of CHB is an established model of immune tolerant CHB and has been used to evaluate therapeutic vaccines and treatments such as TLR ligands CpG (21) and R848 (20, 22) and innate cytokines such as IL-12 (23). Upon liver transduction with AAV-HBV, mice stably express HBsAg for 6–12 months, which induces T- and B-cell immune tolerance to HBV and AAV antigens (13, 17, 24). Indeed, animals in this model fail to induce anti-HBsAg antibodies to CpG-adjuvanted commercial vaccine administered 3–4 weeks after AAV-HBV transduction (17). Michler and colleagues (13) demonstrated a therapeutic response in mice treated with a heterologous protein prime/MVA-boost hepatitis B vaccine (*TherVacB*). Mice were immunized twice with 10 μg of each of HBsAg and HBcAg protein formulated with various adjuvants, followed by 3 × 10^7^ infectious units of recombinant MVA virus expressing HBV S or core at a 2-week interval. This strategy induced anti-HBs antibody and CD4 T-cell–dependent control of infection in mice with HBsAg levels at 10^3^ IU/mL or lower (13, 25).

In our AAV-HBV model, we found that mice with HBsAg below 10^3^ IU/mL responded to therapeutic vaccination with Tetra-3 and controlled HBsAg, whereas mice with HBsAg above 10^3^ IU/mL had no reduction in HBsAg or HBeAg. Clinically, HBsAg levels can vary widely among patients during the course of CHB infection; hence, it is likely that many, if not most, patients have had historical peak serum HBsAg greater than a threshold of 10^3^ IU/mL (26). The high-to-low treatment model using siRNA may more accurately reflect the dynamics of HBsAg changes observed in chronic HBV.

In our Hi-to-Lo–titer model, animals with HBsAg levels of 10^4^–10^5^ IU/mL 1 month post–AAV-HBV transduction were first treated with siRNA to lower HBsAg levels and, 2 weeks later, co-dosed with siRNA and Tetra-3, a tetracistronic self-amplifying RNA (replicon) HBV therapeutic vaccine encoding HBV proteins core, pol, preS2.S, and preS1 (14). Mice transduced with a lower AAV-HBV inoculum, resulting in peak HBsAg serum titer of 10^2^ IU/mL, effectively responded to therapeutic vaccination with detectable immune response and control of HBsAg, whereas high-titer AAV-HBV–transduced animals could not control HBsAg following vaccination even after siRNA treatment lowered HBsAg antigen levels. These data suggest that peak viral titers are sufficient to induce tolerance that persists even after siRNA-mediated suppression of HBsAg prior to therapeutic vaccination. Here, serum HBsAg was reduced by siRNA for only 2 weeks prior to therapeutic vaccination, and we have previously administered siRNA as long as 6 weeks prior to therapeutic vaccination with similar lack of efficacy in the Hi-to-Lo mouse model (unpublished); however, it is possible that a longer duration of siRNA-mediated suppression of HBsAg titers may reduce immune tolerance to therapeutic vaccination.

Recently, similar experiments reduced HBV replication and gene expression to achieve control through a sequential combination of siRNA and therapeutic vaccination in mice persistently transduced and expressing HBsAg (10^3^ IU/mL). Of note, neither induction of neutralizing antibodies reducing circulating HBsAg levels nor siRNA/shRNA-mediated knockdown of HBV gene expression alone induced T-cell immunity (25). This model has not examined the effect in mice with higher HBsAg titers at or above 10^4^ IU/mL, which may be more reminiscent of the majority of untreated CHB patients (7, 27).

A strong HBV-specific CD8 T-cell response is required for HBV clearance in acute infection (28) but in chronic HBV the T-cell response is dysfunctional and is not fully restored by nucleoside analogs (8). HBV-specific T cells in chronic infection have a transcriptional program consistent with exhausted T cells circulating in the periphery and express multiple inhibitory receptors including PD1, LAG3, and Tim3 (29, 30). Both the duration of infection as well as antigen load correlate with loss of HBs- and HBc-specific CD8 T cells, where PDL1 blockade *ex vivo* restored functionality in low HBsAg level patients but not from high antigen levels (31). In addition, HBsAg-specific T cells have the highest PD1 expression, suggesting a more severely exhausted phenotype prone to deletion (8, 32).

Similarly, in our Hi-to-Low AAV-HBV mouse model, we found that induction of HBsAg-specific T cells in response to therapeutic vaccination with Tetra-3 was inversely proportional to serum HBsAg titers, with the highest number of HBsAg-specific T cells induced in naïve mice, a moderate number induced in low HBsAg titer mice, and a minimal induction of HBsAg-specific T cells in high HBsAg titer mice. In contrast, induction of Pol-specific T cells in response to Tetra-3 treatment was unaffected by serum HBsAg levels. Liver-resident HBsAg-specific T cells had the highest levels of PD1 expression and produced less IFNγ and TNFα compared to spleen-resident HBsAg-specific T cells and liver- and spleen-resident Pol- or Core-specific T cells. Such epitope shifts and variation in T-cell exhaustion result may reflect stoichiometry of viral particles linked to high levels of HBsAg or differences in tissue distribution and antigen presentation (33, 34).

Immunotherapy with vaccines and checkpoint inhibitors can boost T-cell functions *in vitro* and, therefore, may be used to reinvigorate the impaired HBV-specific T-cell response in CHB patients (29, 35). One aim of our studies was to identify immune pathways/modulators that, in combination with siRNA and TxVx, could control HBsAg in the liver of chronically HBV-transduced mice with high serum HBsAg titers, reflective of a majority of CHB patients. Here, we found that weekly anti-PDL1 antibody therapy did not change the course of the Hi-to-Lo AAV-HBV treatment model. Similarly, Bunse and colleagues (43) saw no improvement in liver T-cell response to therapeutic vaccination in AAV-HBV mice when the therapeutic vaccine was co-dosed with anti-PDL1 antibody. However, co-dosing the therapeutic vaccine with an siRNA targeting PDL1 during the priming dose, but not during the boosting dose, increased the frequency of IFNγ-secreting T cells approximately two-fold and improved the magnitude of HBsAg reduction compared to vaccine alone, suggesting that early liver T-cell response may benefit from anti-PDL1 antibody therapy. The woodchuck model of HBV has also shown benefit of blocking the PD1/PDL1 axis alongside antiviral treatment and vaccination, with anti-PDL1 treatment during vaccination increasing IFNγ production in blood. Overall, these data suggest that anti-PDL1 gives modest increases in HBV-specific T-cell responses, albeit insufficient to change viral/subviral load. From our observations, a number of other co-inhibitory molecules also did not improve the efficacy of therapeutic vaccination when co-dosed with anti-PDL1 antibody therapy, despite previously reported additive effects *in vitro* on HBV+ human peripheral blood mononuclear cells (PBMCs) and efficacy of combinations in chronic LCMV mouse model (18, 31, 36). In contrast, several co-stimulatory treatments—IL-2-Fc, IL-2:S4B6 complex, or IL-15:IL-15Ra-Fc complex—had clear benefit in augmenting HBsAg control and inducing detectable anti-HBs antibodies and improved T-cell responses.

While the mechanism by which co-dosing Tetra-3 HBV therapeutic vaccine with IL-2–Fc, IL-2:S4B6 complex, or IL-15:IL-15Ra–Fc complex in combination with anti-PDL-1 augments control of HBsAg remains to be elucidated, we speculate the specific IL-2 treatments and anti-PDL1 together activate and expand effector CD8 T cells and NK cells in response to vaccination and prevent their exhaustion. Recent advances in γ chain family cytokine biology and engineering have shown that half-life extension and modulating affinity to IL-2Ra are successful strategies to tune IL-2 signaling preferentially to CD8 T cells and reduce dose limiting toxicities (37). IL-2 pre-complexed with anti–IL-2 clone S4B6 preferentially expands both CD8 T cells and NK cells over CD4 Tregs (38). IL-15:IL-15Ra–Fc also activates T cells and NK cells with extended half-life (39). Most recently, IL-2 agonist linked to anti-PD1 monoclonal antibody allowed for IL-2 targeting of exhausted T cells in LCMV and cancer mouse models with reduced dosing, presumably allowing for safer treatment (40). IL-15 fused to PDL1 is also an effective molecule in treatment of mouse colon and liver tumor models (41). This suggests that increasing effector T-cell number with cytokine therapy in combination with relieving tissue suppression of antigen recognition with anti-PDL1 can augment therapeutic vaccination to control chronic HBV infection.

Combination cytokine and anti-PD(L)1 therapy are being investigated in the clinic for cancer immunotherapy with promising results (42). Applying checkpoint inhibitors at appropriate time and dose with therapeutic vaccination remains a viable strategy for CHB patients. A key observation from our study is that the HBV adaptive immune response can be enhanced through targeted intervention with IL-2 or IL-15 agonists. Newly engineered versions of these agonists with greater selectivity and safety are being advanced clinically for other indications. The results from this study may warrant their assessment as important components, in addition to siRNA and therapeutic vaccination that contribute to the FC of CHB.

## Data Availability

The original contributions presented in the study are included in the article/**Supplementary Material**. Further inquiries can be directed to the corresponding author.
