# Supplementary material for: IL-2 and IL-15 augment HBV therapeutic vaccination and PD1 blockade for functional cure in the AAV-HBV mouse model
Source: Front Immunol. 2025 Jul 16;16:1562107. doi: 10.3389/fimmu.2025.1562107 (PMC12307149; doi:10.3389/fimmu.2025.1562107)
Supplement: Supplementary file 1 [file Table1.docx]

Supplementary Material

**Supplementary Figure 1.** **HBV DNA levels in serum at end of study (week 26).** Mice infected with AAV-HBV to establish chronic infection with high, mid and low circulating HBsAg were treated with 4 doses of Tetra-3 (open circles) or vehicle (closed circles) every two weeks. N=5/group shown as individual animals, lower limit of quantification in gray.


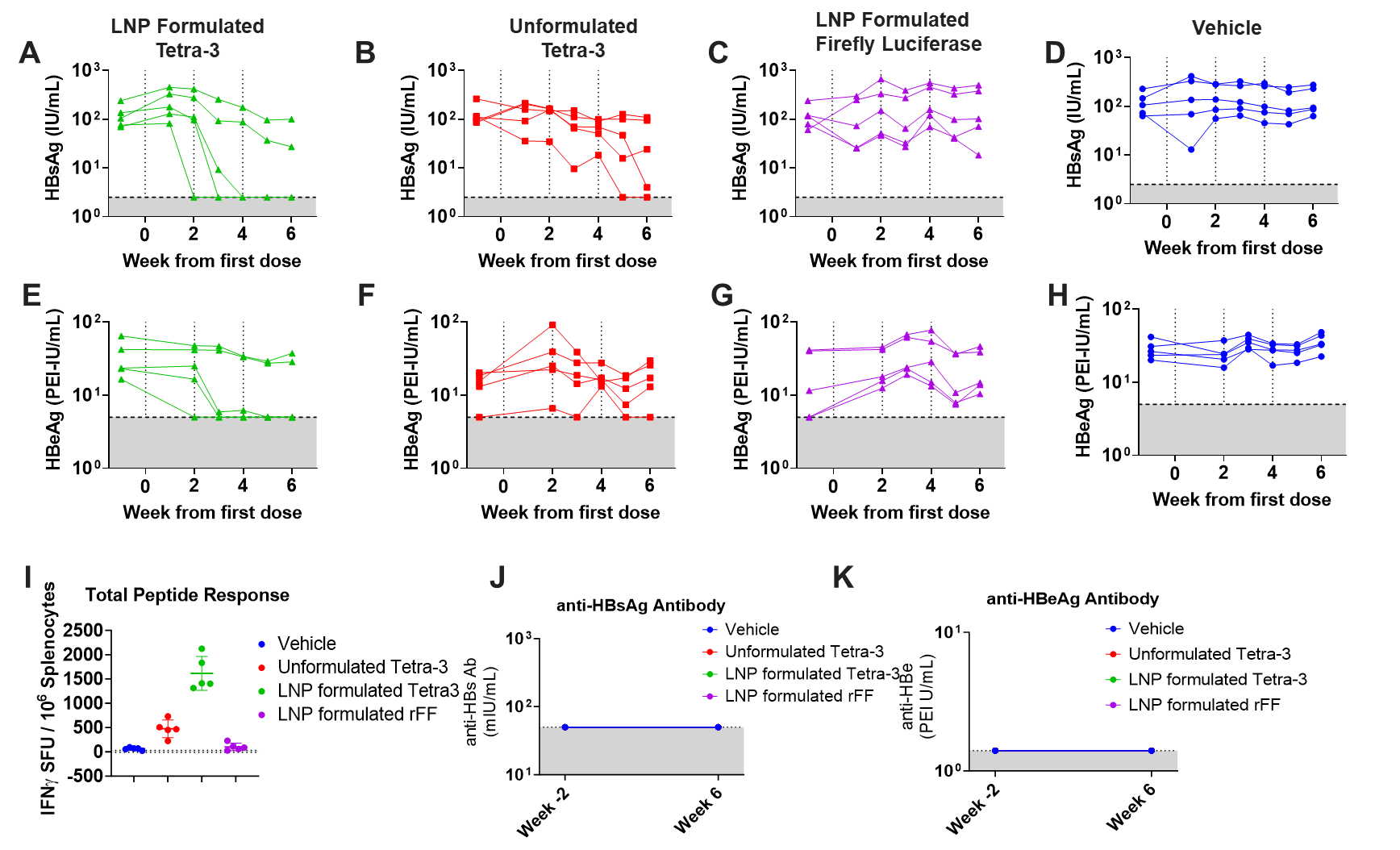


**Supplementary Figure 2.** **Tetra-3 efficacy is mediated by HBV-specific adaptive immune responses.** Mice were inoculated with AAV-HBV to establish mid-titer infection with HBsAg 10^2^-10^3^ IU/mL. for ~7 weeks prior to receiving 3 doses (10 µg) of LNP-formulated Tetra3 (A, E), unformulated Tetra3 (B, F), LNP-formulated firefly luciferase control replicon (C, G) or vehicle (D, H). Serum HBsAg (A, B, C, D) and serum HBeAg (E, F, G, H) concentrations. I) 5 weeks after the last Tetra3 or firefly luciferase replicon dose, IFNγ splenic T cell responses to HBV peptides was quantified by ELISpot. Anti-HBsAg (J) and anti-HBeAg (K) antibody responses two weeks prior to therapeutic vaccination (week -2) and two weeks after the final Tetra3 or firefly luciferase control replicon dose (week 6). N=5/group shown as individual animals, lower limit of quantification in gray.


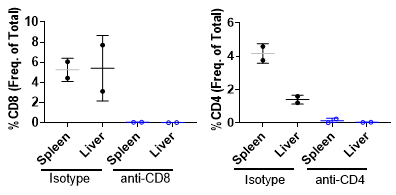


**Supplementary Figure 3.** **T cell dependent control of HBsAg after therapeutic vaccination.** Animals received 100µg anti-CD4, CD8 or isotype control antibody i.p. on days -3 and -1 prior to first Tetra-3 therapeutic vaccination mice i.m.at Day 0 (Figure 3). At day 6 post first dose, the depletion of CD4 and CD8 T cells in spleen and liver tissue was determined in a small subgroup of animals by flow cytometry. N= 2 mice/group shown as individual animals.


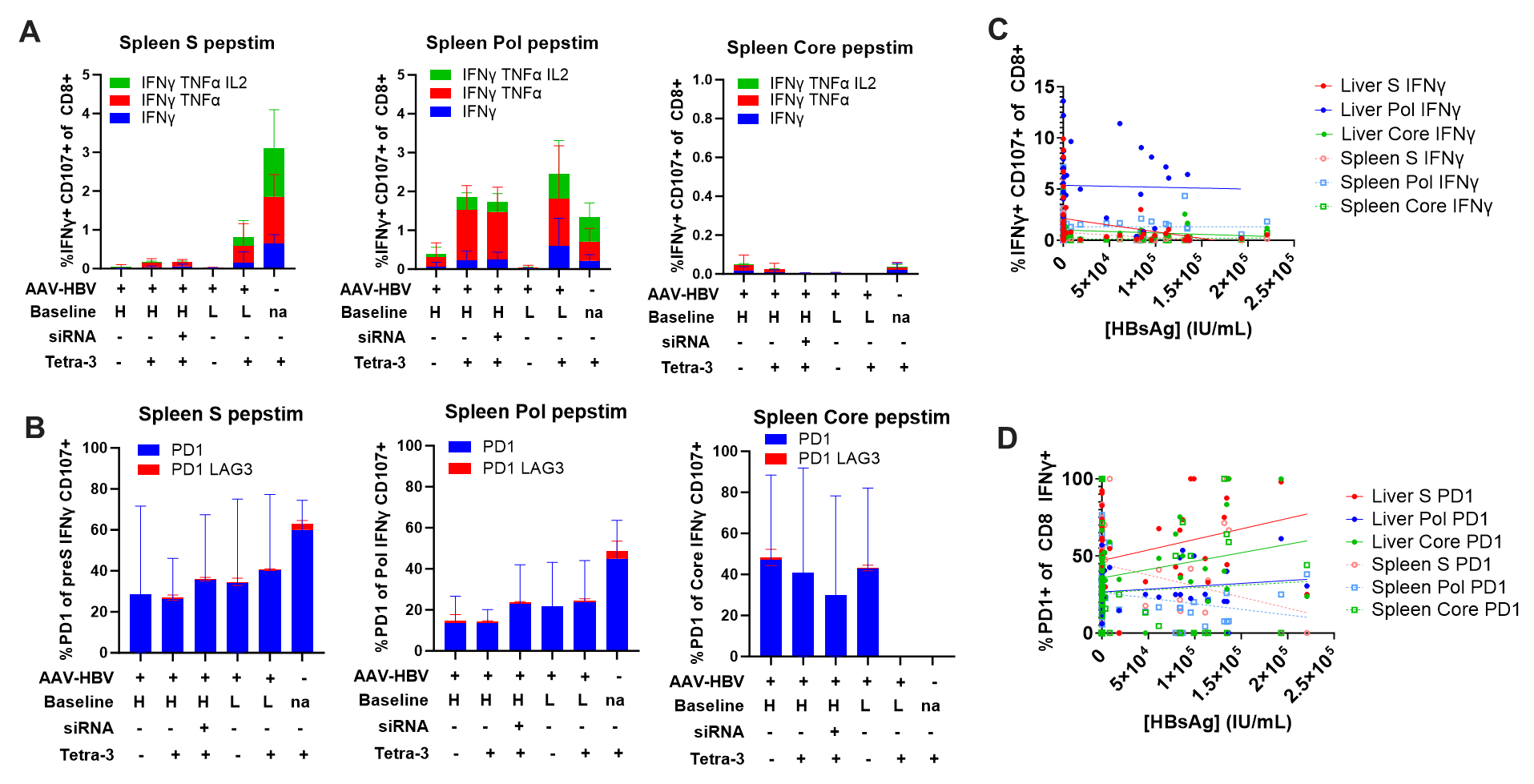


**Supplementary Figure 4.** **High peak HBsAg impairs immunogenicity in spleen and reduces efficacy of therapeutic vaccination.** A) Splenocytes were analyzed for IFNγ, TNF and IL2 by intracellular flow cytometry and graphed as percentage of IFNγ+ CD107A+ CD8+ T cells responding to indicated peptide pools producing IFNγ only (blue) or co-producing TNFα (red) and IL2 (green). B) Splenocytes were analyzed for PD1 expression (blue) and PD1 + LAG3 co-expression (red) by flow cytometry and graphed as a percentage of IFNγ+ CD107A+ T cells responding to indicated peptide pools. C) Correlation of percentage of splenic and liver IFNγ+ CD107A+ CD8+ T cells responding to indicated peptide pools to serum HBsAg concentration at week 8. D) Correlation of percentage of PD1+ IFNγ+ CD8 T cells responding to indicated peptide pools to serum HBsAg concentration at week 8.


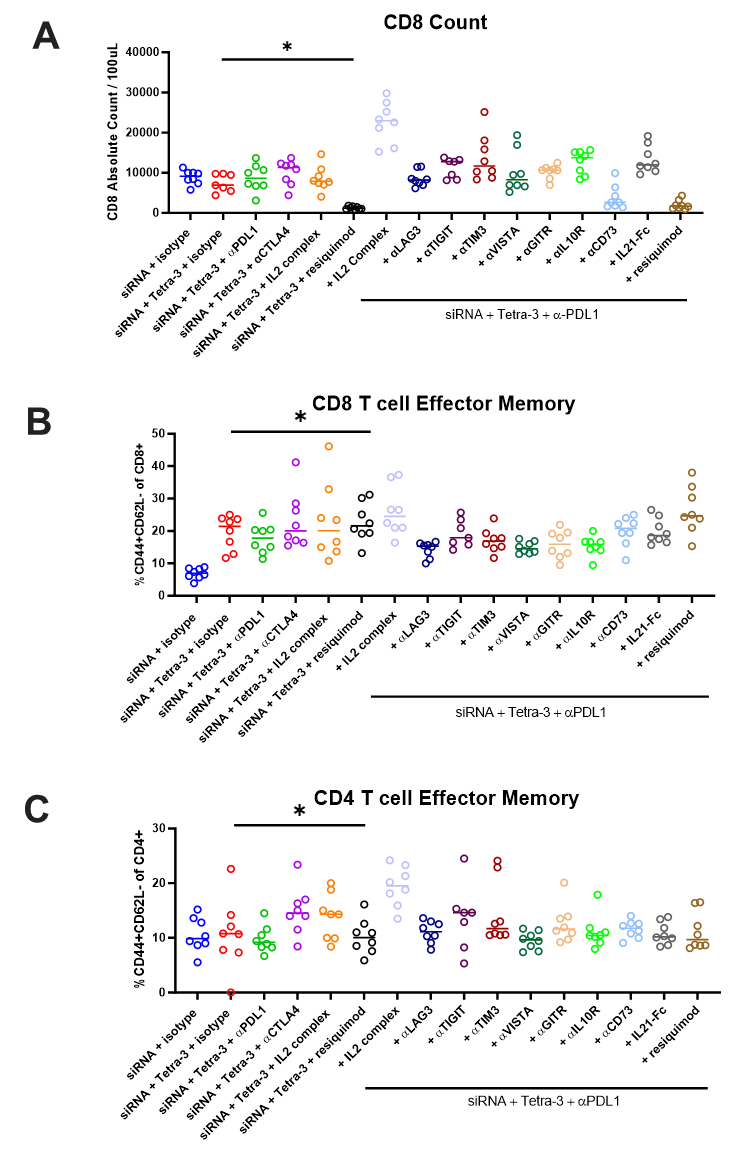


**Supplementary Figure 5. Co-dosing Tetra-3 with anti-PD1 and IL-2 complex significantly increases absolute CD8 T cell count and frequency of effector memory CD8 and CD4 T cells.** CD8 T cell count (A) percent CD8 effector memory T cells (CD44+, CD62L-) (B) and percent CD4 effector memory (CD44+, CD62L-) T cells (C) in peripheral blood at week 12 of mice treated with Tetra-3 co-dosed with anti-PDL1 and IL-2:S4B6 complex.


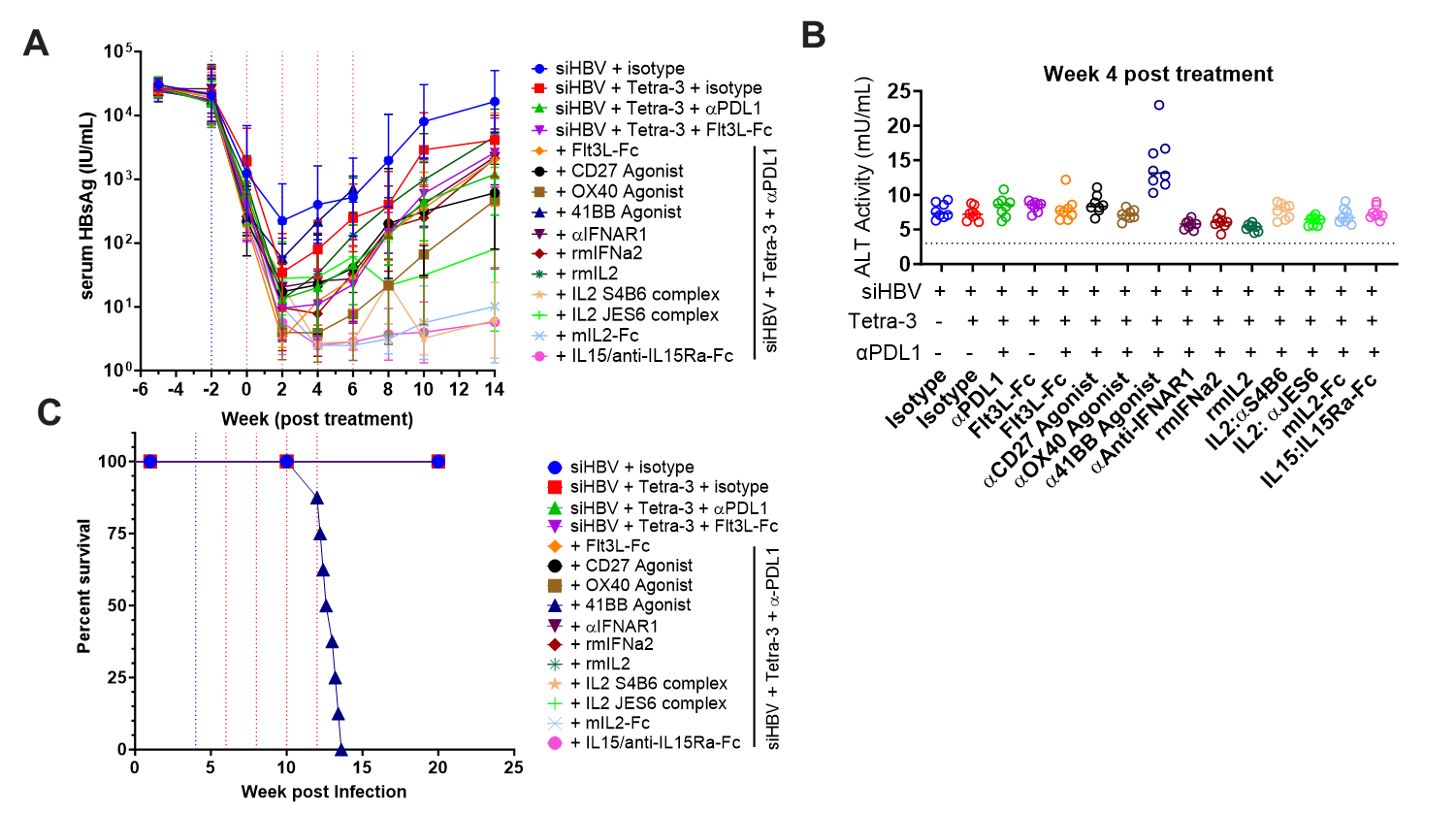


**Supplementary Figure 6.** **Co-stimulation pathway screen for restoration of anti-HBV immune response in high titer infected female mice.** A) Serum HBsAg concentrations in female mice treated with Tetra-3 + anti-PDL1 + immune co-stimulatory agonists. B) Percent survival C) ALT levels at week 4 post treatment.
